# A high eosinophil proportion increases the risk of skin-related adverse events induced by apalutamide in patients with prostate cancer

**DOI:** 10.3389/fimmu.2025.1681734

**Published:** 2025-10-06

**Authors:** Yoshihiko Tasaki, Taku Naiki, Yoshihisa Mimura, Yosuke Sugiyama, Misato Tomita, Toshiharu Morikawa, Takashi Nagai, Rei Unno, Toshiki Etani, Shuzo Hamamoto, Yukihiro Umemoto, Takahiro Yasui, Yoko Furukawa-Hibi

**Affiliations:** ^1^ Department of Clinical Pharmaceutics, Nagoya City University Graduate School of Medical Sciences, Nagoya, Aichi, Japan; ^2^ Department of Nephro-urology, Nagoya City University Graduate School of Medical Sciences, Nagoya, Aichi, Japan; ^3^ Department of Urology, Nagoya City University West Medical Center, Nagoya, Aichi, Japan

**Keywords:** apalutamide, eosinophil, prostate cancer, pruritus, rash

## Abstract

**Background:**

Skin-related adverse events (AEs) induced by apalutamide occur frequently in Japanese patients with prostate cancer. However, biomarkers for predicting these skin-related AEs have not yet been identified. Therefore, this study investigated whether the proportion of eosinophils could serve as a predictive biomarker for skin-related AEs in Japanese patients with prostate cancer treated with apalutamide.

**Methods:**

A total of 109 patients were enrolled in this study. Among them, 79 patients with prostate cancer who received apalutamide were categorized into two groups: the skin AE group (n = 45) and the non-skin AE group (n = 34), based on whether they experienced skin-related AEs of any grade. The eosinophil proportions in baseline samples collected before treatment were then analyzed.

**Results:**

The baseline eosinophil proportion was significantly higher in the skin AE group compared with the non-skin AE group (*P* < 0.05). The optimal cut-off value of the eosinophil proportion for predicting skin-related AEs of any grade was 1.8% (area under the receiver operating characteristic curve [AUC] = 0.768). In multivariate analysis, an eosinophil proportion ≥1.8% was identified as an independent factor associated with skin-related AEs of any grade (odds ratio, 13.3; 95% confidence interval, 3.82–46.4; *P* < 0.05).

**Conclusion:**

The baseline eosinophil proportion may serve as a predictive biomarker for skin-related AEs of any grade in Japanese patients with prostate cancer treated with apalutamide.

## 1 Introduction

Prostate cancer has the highest incidence among all cancers in men (1). Its 5-year survival rate is higher than many other cancers, largely due to advances in therapeutic agents (1). Among these agents, apalutamide, an androgen receptor signaling inhibitor, is a key therapeutic agent for patients with non-metastatic castration-resistant prostate cancer (nmCRPC) and metastatic hormone-sensitive prostate cancer (mHSPC) (2–4). Phase 3 trials demonstrated that patients with nmCRPC or mHSPC treated with apalutamide had significantly longer metastasis-free and progression-free survival than those receiving placebo (2, 3).

Although highly effective, apalutamide is associated with skin-related adverse events (AEs) that are not commonly observed with other androgen receptor signaling inhibitors and occur at a relatively high frequency (2, 3). In phase 3 trials, over 20% of patients experienced skin-related AEs induced by apalutamide (2, 3). Notably, subgroup and integrated analyses from these trials reported that the incidence of skin-related AEs in Japanese patients exceeded 50%, which was higher than that observed in the overall global population (4, 5). Similarly, other Asian populations, such as Chinese and Korean patients, also exhibit a higher incidence of skin-related AEs compared with the overall phase 3 trial populations (6, 7). These findings suggest that skin-related AEs occur at a disproportionately high rate in Asian populations, particularly among Japanese patients (4, 5). However, no practical biomarkers for predicting these events have been reported.

Although the mechanism underlying skin-related AEs remains unclear, case reports have documented eosinophil infiltration into skin tissue and an elevated eosinophil proportion in peripheral blood (8, 9). Therefore, this study investigated whether eosinophil levels could serve as a biomarker for skin-related AEs in Japanese patients with prostate cancer treated with apalutamide.

## 2 Methods

### 2.1 Patient characteristics and data collection

**Figure 1:**
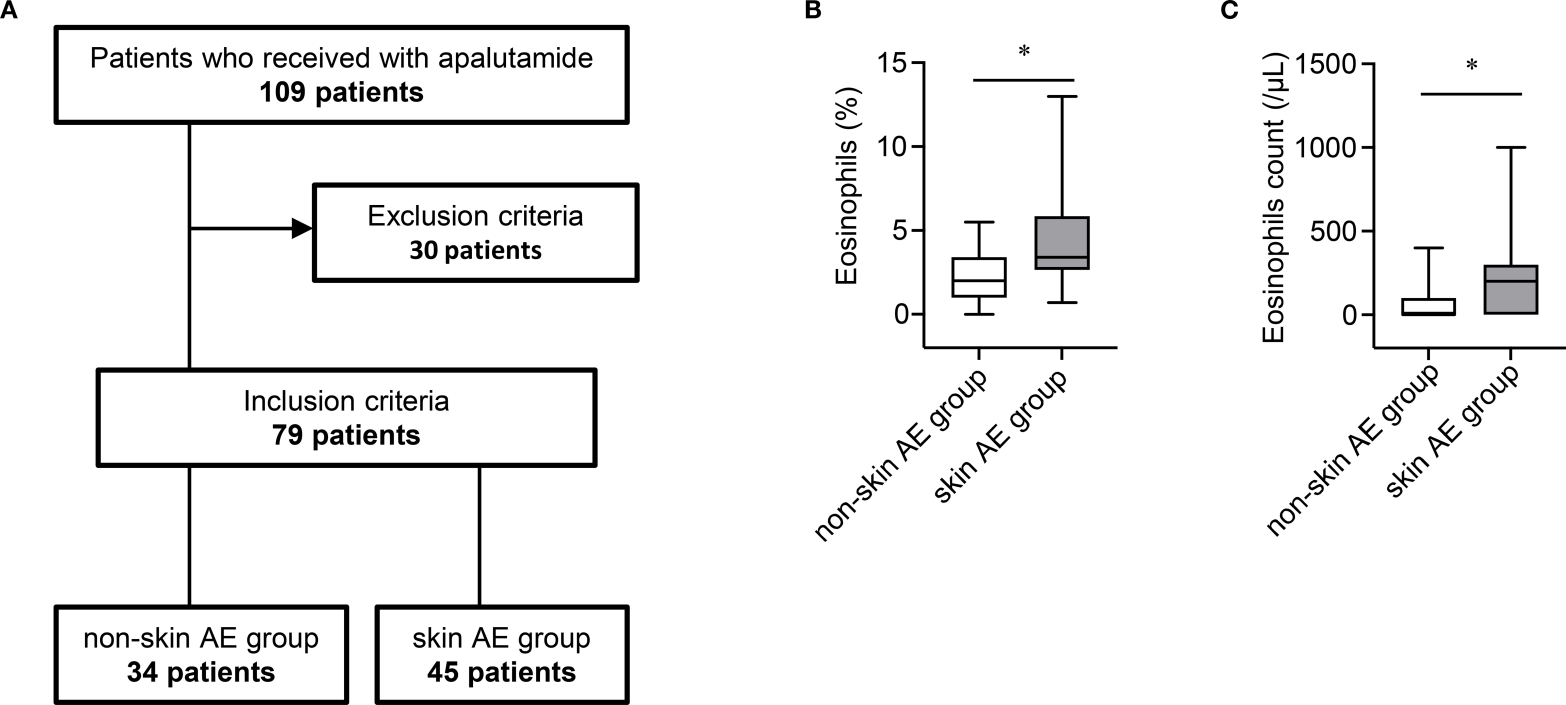
**(A)** Patient enrollment flowchart. **(B)** Boxplot of baseline eosinophil proportions in the non-skin AE group (n = 34) and skin AE group (n = 45). AE, adverse event. *P < 0.05. **(C)** Boxplot of baseline eosinophil counts in the non-skin AE group (n = 34) and skin AE group (n = 45). AE, adverse event. *P < 0.05.

The patient enrollment process is shown in **Figure 1A**. We retrospectively analyzed the medical records and baseline eosinophil proportions (measured prior to apalutamide initiation) of 109 patients with nmCRPC or mHSPC who received apalutamide at Nagoya City University Hospital and Nagoya City University West Medical Center in Japan between January 2019 and March 2025. Thirty patients whose eosinophil proportion was not measured before the initiation of apalutamide were excluded. Consequently, 79 patients were included in the analysis and classified into two groups based on the occurrence of skin-related AEs (rash and pruritus): skin AE group and non-skin AE group. Patient characteristics of excluded (n = 30) and included (n = 79) patients are summarized in **Supplementary Table S1**. The severity of skin-related AEs was graded according to the Common Terminology Criteria for Adverse Events (version 5.0).

### 2.2 Evaluation of eosinophil proportions and the occurrence of skin-related AEs

Receiver operating characteristic (ROC) curves were used to determine the optimal cut-off values for eosinophil proportion, eosinophil count, body weight, and body surface area associated with skin-related AEs of any grade (**Supplementary Figure S1**). Risk factors for skin-related AEs of any grade were then evaluated using univariate and multivariate logistic regression analyses.

### 2.3 Statistical analysis

A *P* < 0.05 was considered statistically significant. Differences in the quantified data of the groups were compared using the t-test. Fisher’s exact test and one-way analysis of variance were applied to assess differences in patient characteristics. All statistical analyses were conducted using GraphPad Prism 9 software and EZR (Saitama Medical Center, Jichi Medical University, Saitama, Japan) (10).

## 3 Results

### 3.1 Patient characteristics and safety information

Patient characteristics are summarized in **Table 1**. Among the 79 patients included in this study, 34 patients (43.0%) were classified into the non-skin AE group and 45 patients (57.0%) into the skin AE group. Age, body weight, body surface area, prostate-specific antigen level, primary Gleason score, distribution of nmCRPC and mHSPC cases, metastasis sites (bone, liver, lung, lymph nodes), and median treatment duration did not differ significantly between the two groups. However, the median relative dose intensity of apalutamide differed between the non-skin AE and skin AE groups.

**Table 1:** Clinical features of the study patients.

Characteristics, n (%)	Non-skin AE group	Skin AE group	*P* value
	34 (100)	45 (100)	
Age, median (range)	74.5 (60–92)	75 (64–87)	0.27
Body weight, median (range)	59.5 (42.8–86.4)	62.0 (44.5–82.4)	0.23
Body surface area, m^2^, median (range)	1.64 (1.40–1.92)	1.67 (1.40–2.02)	0.57
PSA, ng/mL, median (range)	18.4 (0.04–2143)	26.31 (0.20–6535)	0.70
Primary Gleason score, n (%)			0.71
3 + 4	1 (2.9)	0 (0.0)	
4 + 3	2 (5.9)	1 (2.2)	
4 + 4	10 (29.4)	14 (31.1)	
4 + 5	10 (29.4)	16 (35.6)	
5 + 4	3 (8.8)	5 (11.1)	
5 + 5	6 (17.6)	4 (8.9)	
Unknown	2 (5.9)	5 (11.1)	
Treatment target			0.61
nmCRPC	26 (76.5)	32 (71.1)	
mHSPC	8 (23.5)	13 (28.9)	
Metastasis site, bone	23 (67.6)	27 (60.0)	0.63
Metastasis site, liver	1 (2.9)	1 (2.2)	1.00
Metastasis site, lung	5 (14.7)	4 (8.9)	0.48
Metastasis site, lymph node	17 (50.0)	22 (48.9)	1.00
Total apalutamide treatment period, day, median (range)	425 (19–1654)	407 (4–1885)	0.81
Relative dose intensity of apalutamide, %, median (range)	100 (67.5–100)	84.5 (26.2–100)	<0.05

AE, adverse event; mHSPC, metastatic hormone-sensitive prostate cancer; nmCRPC, non-metastatic castration-resistant prostate cancer; PSA, prostate-specific antigen.

Safety information for apalutamide is summarized in **Table 2**. In total, 45 patients (57%) experienced skin-related AEs. Of these 45 patients, 36 (80.0%), 7 (15.6%), and 2 (4.4%) experienced grades 1, 2, and 3 AEs, respectively. The median time to onset of skin-related AEs was 55 days (range, 6–889 days). Furthermore, 11 (24.4%) events occurred within the first 30 days of treatment, 11 (24.4%) occurred between 31 and 60 days, 16 (35.6%) occurred between 61 and 120 days, and 5 (11.1%) occurred at 121 days or later.

**Table 2:** Characteristics of skin-related adverse events.

Characteristics, n (%)	Skin AE group (n = 45 patients)
Severity of skin-related AEs, n (%)	
Grade 1	36 (80.0)
Grade 2	7 (15.6)
Grade 3	2 (4.4)
Duration of skin-related AEs, day, median (range)	55 (6–889)
Time from initiation of apalutamide to skin-related AEs, n (%)	
0–30 days	11 (24.4)
31–60 days	11 (24.4)
61–120 days	16 (35.6)
≥121 days	5 (11.1)
Not evaluated	2 (4.4)

AE, adverse event.

### 3.2 Evaluation of associations between eosinophils and the risk of skin-related AEs

Because approximately 20% of patients experienced skin-related AEs within the first 30 days of treatment, identifying the risk of AE occurrence in the early treatment phase with apalutamide is clinically important. Therefore, we examined whether the baseline eosinophil proportion was associated with the occurrence of skin-related AEs. The baseline eosinophil proportion was significantly higher in the skin AE group than in the non-skin AE group (mean: 4.3% vs. 2.1%; *P* < 0.05) (**Figure 1B**). Similarly, the baseline eosinophil count was significantly higher in the skin AE group than in the non-skin AE group (mean: 208.9/μL vs. 67.6/μL; *P* < 0.05) (**Figure 1C**). The optimal cut-off value of the baseline eosinophil proportion associated with skin-related AEs of any grade was 1.8% (area under the receiver-operating characteristic curve = 0.768; specificity = 0.588; sensitivity = 0.911) (**Supplementary Figure S1A**). In both univariate and multivariate analyses, a baseline eosinophil proportion ≥1.8% was an independent factor associated with skin-related AEs of any grade (**Table 3**). Multicollinearity was excluded in the multivariate analysis (Variance Inflation Factor: age, 1.05; body weight, 2.96; body surface area, 3.01; eosinophil, 1.01).

**Table 3:** Univariate and multivariate logistic regression analyses of risk factors for skin-related adverse events of any grade.

	Univariate			Multivariate		
	OR	95% CI	*P* value	OR	95% CI	*P* value
Age 75 years or older	1.50	0.61–3.68	0.37	1.30	0.43–3.90	0.63
Body weight ≥61.1 kg	1.79	0.72–4.40	0.20	1.03	0.16–6.43	0.97
Body surface area ≥1.633 m^2^	2.00	0.80–4.99	0.13	1.55	0.23–10.1	0.64
Baseline proportion of eosinophils ≥1.8%	14.6	4.27–50.3	<0.05	13.3	3.82–46.4	<0.05

CI, confidence interval; OR, odds ratio.

## 4 Discussion

In the present study, we found that a high eosinophil proportion was associated with approximately 13-fold increased risk of skin-related AEs, suggesting that eosinophil proportion prior to treatment may serve as a potential biomarker for apalutamide-induced skin-related AEs in patients with nmCRPC and mHSPC.

Skin-related AEs are a well-recognized adverse effect in patients with nmCRPC and mHSPC receiving apalutamide. In the SPARTAN study, 23.8% of patients with nmCRPC experienced skin-related AEs (2). Similarly, in the TITAN study, 27.1% of patients with mHSPC developed skin-related AEs (3). In these clinical trials, the median time to first skin-related AE was 82 days in patients with nmCRPC and 80.5 days in those with mHSPC (2, 3). By contrast, in our study, 57% of patients experienced skin-related AEs, with a median time to onset of 55 days. Thus, both the incidence rate and the onset of skin-related AEs in our study were higher and earlier than those observed in previous clinical trials. Uemura et al. conducted an integrated analysis and reported skin-related AEs in 51.5% of patients, which is consistent with our findings (5). Their integrated analysis also showed that the median time to the onset of skin-related AE was 66 days, and that skin-related AEs occurred faster in Japanese patients compared with the overall global population (5). Taken together, both our findings and integrated analyses indicate that skin-related AEs are particularly common among Japanese patients and tend to occur earlier during treatment.

Notably, Perez-Ruixo et al. reported a statistically significant association between skin-related AEs and increased apalutamide exposure (11). Uemura et al. further reported that apalutamide exposure levels of Japanese patients are higher than in non-Japanese patients (4, 5); however, the greater incidence of skin-related AEs among Japanese patients could not be fully explained by these higher apalutamide exposure levels (4, 5). In addition, previous studies have reported associations between skin-related AEs and age, body surface area, and body weight (12, 13). In the present study, the relative dose intensity was higher in the non-skin AE group than in the skin AE group (**Table 1**). Furthermore, age, body weight, and body surface area were not identified as risk factors for skin-related AEs (**Table 3**). Therefore, the detailed mechanisms underlying the higher incidence of skin-related AEs in Japanese patients with nmCRPC and mHSPC remain unclear. However, our findings and prior integrated analyses suggest that physicians should carefully adjust the dose of apalutamide according to the presence and severity of skin-related AEs, and ensure appropriate management, particularly in Japanese patients. We confirmed that baseline eosinophil proportion was associated with the risk of skin-related AEs. Because eosinophil levels can be easily measured in clinical practice, this approach may facilitate more effective management of skin-related AEs.

The detailed mechanism by which apalutamide causes skin-related AEs has not been fully clarified. However, one study investigated this mechanism from the perspective of the compound’s chemical structure (14). Ji et al. noted that although apalutamide and enzalutamide are structurally similar, they exhibit different AEs profiles, and they examined whether the unique structure of apalutamide could explain the occurrence of skin-related AEs (14). Their findings suggested that the 2-cyanopyridine residue in apalutamide may react with cysteine residues in proteins to form haptens. This hapten formation triggers activation of CD4^+^ T cells, CD8^+^ T cells, and B cells (14). Supporting this mechanism, two case reports on apalutamide-induced skin-related AEs documented lymphocyte infiltration in skin tissue (8, 9). Thus, lymphocyte activation caused by the unique structure of apalutamide may contribute to skin-related AEs. In the present study, we demonstrated an association between skin-related AEs and eosinophil levels in patients treated with apalutamide. Previously, we focused on immune checkpoint inhibitors (ICIs) when examining the relationship between eosinophils and treatment-related AEs, and we demonstrated that a high eosinophil proportion increases the risk of AEs in patients receiving ICIs (15–17). The association between ICIs and eosinophils is biologically plausible, given the immunostimulatory effects of ICIs. Mechanistic studies have shown that activation of CD4^+^ cells by ICI treatment leads to secretion of interleukin-5, which promotes eosinophil production in the bone marrow and their accumulation in peripheral blood (18). Although the direct association between apalutamide and eosinophils remains unclear, previous reports suggest that apalutamide and ICIs may share similar pathways of immune activation (14, 18). Several studies that focused on skin tissue affected by skin-related AEs caused by apalutamide have reported eosinophil and lymphocyte infiltration of skin tissue, along with increased eosinophil levels in peripheral blood (8, 9). Moreover, drug eruptions such as erythema multiform are associated with elevated eosinophil levels in both peripheral blood and skin lesions (13, 14). During drug eruptions, the production of cytokines and chemokines such as IL-5 and IL-3, which promote eosinophil differentiation in the bone marrow, is increased, leading to their accumulation in peripheral blood and infiltration into skin lesions (19, 20). A more detailed analysis of the relationship between eosinophils and apalutamide-induced skin-related AEs is warranted. Taken together with previous studies—such as lymphocyte activation by apalutamide, lymphocyte-mediated promotion of eosinophil production, and eosinophil/lymphocyte infiltration into skin tissue—our results are supported by existing evidence.

This study has some limitations. First, patient selection bias could not be controlled because of its retrospective design. Second, the sample size was small. Third, baseline eosinophil data were missing for 30 patients before treatment. Therefore, our results should be validated in larger, independent cohorts.

In conclusion, our findings may have clinical applicability and suggest that the pretreatment eosinophil proportion could serve as a useful biomarker for apalutamide-induced skin-related AEs in patients with nmCRPC and mHSPC.

## Data Availability

The raw data supporting the conclusions of this article will be made available by the authors, without undue reservation.
