# Supplementary material for: A high eosinophil proportion increases the risk of skin-related adverse events induced by apalutamide in patients with prostate cancer
Source: Front Immunol. 2025 Oct 6;16:1681734. doi: 10.3389/fimmu.2025.1681734 (PMC12536007; doi:10.3389/fimmu.2025.1681734)
Supplement: Supplementary file 1 [file Presentation1.pptx]

## Slide 1
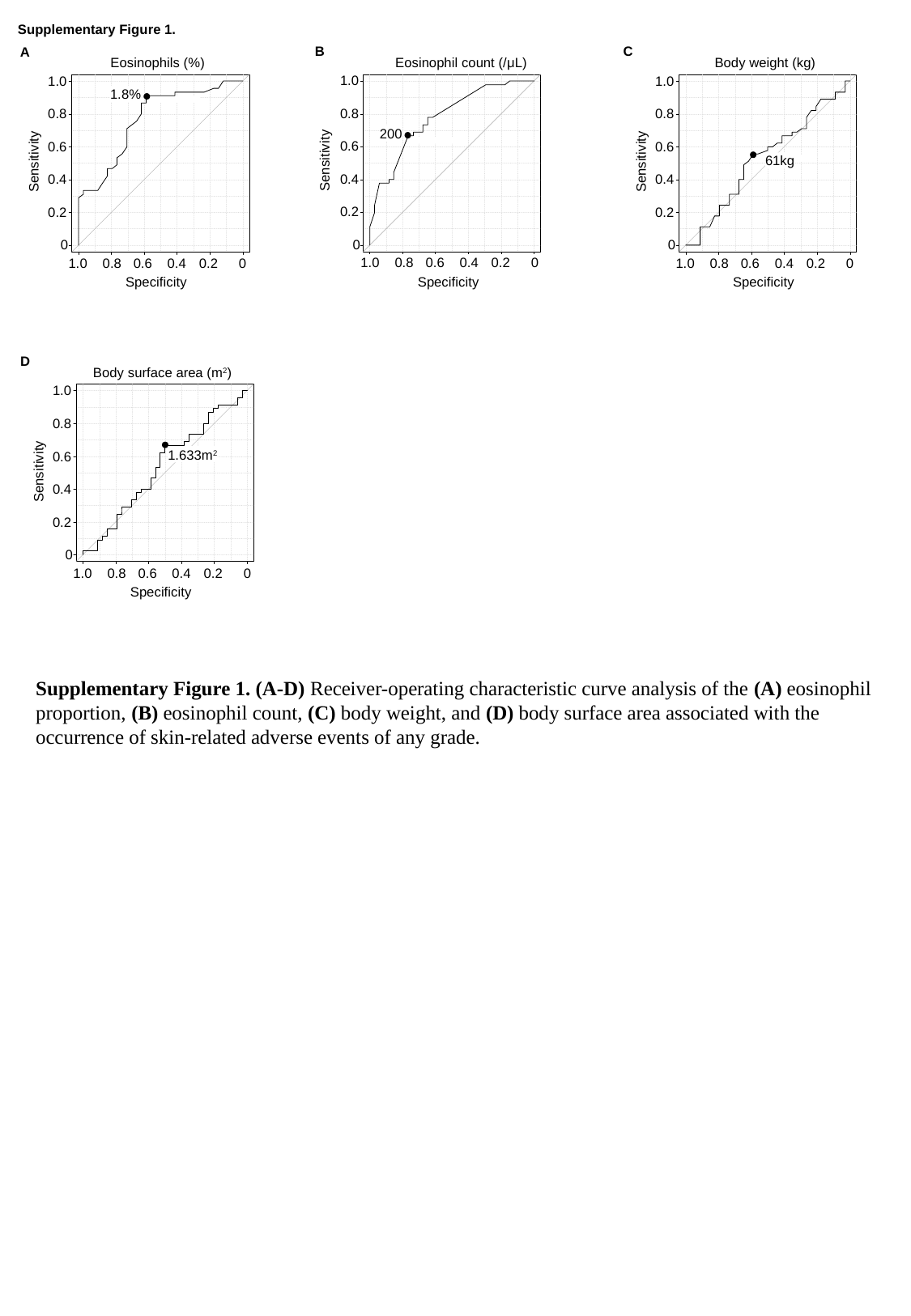

Supplementary Figure 1.
B
C
A
Eosinophil count (/μL)
Eosinophils (%)
Body weight (kg)
1.0
1.0
1.0
1.8%
0.8
0.8
0.8
200
0.6
0.6
0.6
Sensitivity
Sensitivity
Sensitivity
61kg
0.4
0.4
0.4
0.2
0.2
0.2
0
0
0
1.0
0.8
0.6
0.4
0.2
0
1.0
0.8
0.6
0.4
0.2
0
1.0
0.8
0.6
0.4
0.2
0
Specificity
Specificity
Specificity
D
Body surface area (m2)
1.0
0.8
1.633m2
0.6
Sensitivity
0.4
0.2
0
1.0
0.8
0.6
0.4
0.2
0
Specificity
Supplementary Figure 1. (A-D) Receiver-operating characteristic curve analysis of the (A) eosinophil proportion, (B) eosinophil count, (C) body weight, and (D) body surface area associated with the occurrence of skin-related adverse events of any grade.
