# Supplementary material for: A high eosinophil proportion increases the risk of skin-related adverse events induced by apalutamide in patients with prostate cancer
Source: Front Immunol. 2025 Oct 6;16:1681734. doi: 10.3389/fimmu.2025.1681734 (PMC12536007; doi:10.3389/fimmu.2025.1681734)
Supplement: Supplementary file 2 [file Table1.docx]

Supplementary Material

# Supplementary Table

**Supplementary Table 1. Clinical features** **of excluded and included patients**

|  | Excluded patients | Included patients | *P* value |
| --- | --- | --- | --- |
| Characteristics, n (%) | 30 (100) | 79 (100) |  |
| Age, median (range) | 77 (60–89) | 75 (60–92) | 0.57 |
| Body weight, median (range) | 60.5 (32.0–79.0) | 60.5 (42.8–86.4) | 0.70 |
| Body surface area, m^2^, median (range) | 1.66 (1.20–1.85) | 1.66 (1.40–2.02) | 0.89 |
| PSA, ng/mL, median (range) | 6.21 (0.12–3516) | 24.76 (0.04–6535) | 0.15 |
| Primary Gleason score, n (%) |  |  | 0.28 |
| 3+3 | 2 (6.7) | 0 (0.0) |  |
| 3+4 | 0 (0.0) | 1 (1.3) |  |
| 4+3 | 2 (6.7) | 3 (3.8) |  |
| 4+4 | 11 (36.7) | 24 (30.4) |  |
| 4+5 | 5 (16.7) | 26 (32.9) |  |
| 5+4 | 4 (13.3) | 8 (10.1) |  |
| 5+5 | 3 (10.0) | 10 (12.7) |  |
| Unknown | 3 (10.0) | 7 (8.9) |  |
| Treatment target |  |  | <0.05 |
| nmCRPC | 15 (50.0) | 58 (73.4) |  |
| mHSPC | 15 (50.0) | 21 (26.6) |  |
| Metastasis site, bone | 11 (33.3) | 50 (63.3) | <0.05 |
| Metastasis site, liver | 1 (3.3) | 2 (2.5) | 1.00 |
| Metastasis site, lung | 3 (10.0) | 9 (11.4) | 1.00 |
| Metastasis site, lymph node | 11 (36.7) | 39 (49.4) | 0.28 |
| The occurrence of skin-related AEs; Yes | 17 (56.7) | 45 (57.0) | 1.00 |

AE, adverse event; mHSPC, metastatic hormone-sensitive prostate cancer; nmCRPC, non-metastatic castration-resistant prostate cancer; PSA, prostate-specific antigen.
